# Minimally Invasive Surgery Oblique Lumbar Interbody Debridement and Fusion for the Treatment of Lumbar Spondylodiscitis

**DOI:** 10.1111/os.12711

**Published:** 2020-06-10

**Authors:** Bingjin Wang, Chao Chen, Wenbin Hua, Wencan Ke, Saideng Lu, Yukun Zhang, Xianlin Zeng, Cao Yang

**Affiliations:** ^1^ Department of Orthopaedics, Union Hospital, Tongji Medical College Huazhong University of Science and Technology Wuhan China

**Keywords:** Debridement, Lumbar spondylodiscitis, Mis‐OLIF, Oblique lumbar interbody fusion

## Abstract

**Objective:**

To evaluate the efficacy and feasibility of minimally invasive oblique lumbar interbody debridement and fusion for the treatment of conservatively ineffective lumbar spondylodiscitis.

**Methods:**

This is a retrospective study. Between December 2016 and November 2017, a total of 14 consecutive patients (eight males and six females, with an average age of 49.1 years, range from 42 to 74 years) with single‐level lumbar spondylodiscitis were included in the study. The inclusion criteria include single‐level spondylodiscitis without spinal deformity or epidural abscess, ineffective conservative treatment (continuously aggravated clinical symptoms and uncontrollable infective symptoms treated with antibiotics for more than 6 weeks), minimally invasive oblique lumbar interbody fusion surgery (Mis‐OLIF) and iliac graft for the treatment of lumbar spondylodiscitis, and postoperative follow‐up >12 months. Each patient was treated Mis‐OLIF. Clinical outcomes including demographic characteristics, erythrocyte sedimentation rate (ESR), C‐reactive protein (CRP), visual analog scale (VAS), the Oswestry Disability Index (ODI), American Spinal Injury Association neurological classification, and lordotic angle were analyzed.

**Results:**

The infectious levels included L1/2 (one patient), L2/3 (two patients), L3/4 (eight patients), and L4/5 (three patients). The pathogens found in these patients included *Staphylococcus aureus* (5), brucellosis (6), and enterobacterium (2). The pathogen was undefined in one patient. The mean duration of the surgery, mean blood loss, and mean follow‐up were 89.3 ± 17.5 min, 155.0 ± 49.4 mL, and 16.8 ± 4.2 months, respectively. The ESR and CRP decreased after Mis‐OLIF and antibiotic administration. The average preoperative VAS score was 6.9 ± 0.9, then decreased to 3.0 ± 1.0 (t = 14.18, *P* < 0.001) and 0.6 ± 0.7 (t = 20.68, *P* < 0.001) before discharge and at final follow‐up, respectively. The average preoperative ODI score was 58.4 ± 13.0, then decreased to 28.3 ± 6.1 (t = 18.6, *P* < 0.001) and 8.0 ± 4.6 (t = 22.7, *P* < 0.001) before discharge and at final follow‐up, respectively. None of the patients developed postoperative ileus, vascular injury, nerve injury, and ureteral injury. One patient suffered incision‐related complication that healed by debridement and dressing change. One patient developed subsidence of autologous iliac bone before discharge and achieved complete bony fusion after staying in bed and fixing it with a brace at 3 months follow‐up. All patients achieved bony fusion at final follow‐up.

**Conclusion:**

Mis‐OLIF without anterior or posterior instrumentation and iliac graft is an effective and viable approach for the treatment of conservatively ineffective lumbar spondylodiscitis without spinal deformity or epidural abscess.

## Introduction

Spondylodiscitis is defined as an infection of the intervertebral disc and vertebral body. The incidence of spinal spondylodiscitis has increased over the period 1995–2008, and the elderly have the highest risk of suffering from spondylodiscitis^1^. Gram‐positive bacteria have been identified in most patients^2^, ^3^. Nonspecific manifestations in patients with lumbar bacterial spondylodiscitis were detected, and general symptoms included local percussion pain, pain induced by exertion, fever, lack of appetite, and fatigue^4^. Effective radiological methods for the diagnosis of spondylodiscitis include X‐ray, computed tomography (CT), and magnetic resonance images (MRI). The diagnosis of spondylodiscitis is based not only on clinical symptoms and radiological findings but also on laboratory examinations, such as erythrocyte sedimentation rate (ESR) and C‐reactive protein (CRP), and blood culture, especially in patients with persistent or intermittent high fever. Moreover, surgical biopsy is the most effective and adequate method to detect the pathogen^5^. However, the positive rate in the bacterial culture is still low by surgical biopsy or open surgery^6^.

No treatment consensus has yet been reached due to the heterogeneity of spondylodiscitis. Furthermore, diagnose with delay and inaccuracy and inadequate treatment may result in devastating complications of spondylodiscitis^7^. The recommendation of conservative or surgical treatment depends on the clinical symptoms and radiological findings. It was summarized that antibiotic treatment for 6 weeks was sufficient for most patients with non‐specific spondylodiscitis^4^, ^5^. The majority of patients with spondylodiscitis can be cured by conservative treatment, but approximately 10%–20% of the patients may require surgical treatment because of spinal instability, intraspinal abscess, neurological deficits, and ineffective conservative treatment^4^, ^8^. The purpose of surgical intervention was to maintain spinal stabilization, abscess debridement, and release of nerve roots or spinal cord compression^2^, ^4^. Early surgery with antibiotic treatment of spondylodiscitis could achieve better prognosis and reduce relative complications^9^. Surgical intervention includes less invasive techniques (percutaneous endoscopic discectomy and drainage and CT‐guided percutaneous puncture and drainage) and open surgical techniques, including one‐ or two‐stages anterior, posterior, and combined approaches, with or without instrumentation^10^.

Percutaneous endoscopic discectomy and drainage or CT‐guided percutaneous puncture and drainage and antibiotic are safe procedures for early‐stage spinal spondylodiscitis^11^. However, this kind of less invasive surgery is not suitable for patients with spondylodiscitis associated with spine instability or kyphotic deformity caused by extensive bony destruction and epidural abscesses resulting in neurologic dysfunction. Open surgical techniques are still the standard for patients with spondylodiscitis with kyphotic deformity or spine instability and epidural abscesses. The anterior approach with fixation and fusion achieves better clinical results than the posterior fixation^12^. Nonetheless, the anterior approach has the potential for visceral and vascular injury^13^. In addition, for patients who suffer single‐level spondylodiscitis with spine instability caused by bony destruction but without obvious spinal deformity or epidural abscess, open surgical techniques with internal fixation may result in longer operation time, more blood loss, and more obvious tissue trauma. Less invasive surgery with effective results is needed for those patients.

Oblique lumbar interbody fusion (OLIF) has been widely used in lumbar disease and OLIF surgical approach has the potential advantage over conventional anterior approach^14^, ^15^. Minimally invasive oblique lumbar interbody fusion (Mis‐OLIF) surgery gradually became an approach for spinal fusion in the treatment of degenerative lumbar diseases^16^. It was indicated that the OLIF for degenerated lumbar spondylolisthesis achieved satisfied radiographic evaluation and indirect decompression^17^. Use of OLIF technique with a minimally invasive expandable retractor allows for accessing the lumbar anterior column via the oblique retroperitoneal intermuscular space. Moreover, OLIF has been proved to be a safe surgical procedure to manage the lesions at level of L1‐S1^16^, ^18^. When compared to lateral lumbar interbody fusion, OLIF mitigates the complications related to psoas and lumbosacral muscles^18^. In terms of the learning curve, if the practicing spine surgeon could pay close attention to detail, the potential of complication could be reduced to a minimum^16^. Compared with traditional posterior fusion surgeries, several advantages of reduced operation time, tissue trauma, and blood loss have been reported^16^. Considering the advantages of OLIF, Mis‐OLIF strategy may become an effective surgical approach to treat lumber spondylodiscitis. It has been reported that OLIF combined with posterior internal fixation is effective and safe for single‐level spondylodiscitis after ineffective conservative treatment^19^. However, the internal fixation may not be necessary for patients with single‐level lumbar spondylodiscitis without spinal deformity or epidural abscess. The OLIF approach without internal fixation used to treat single‐level lumbar spondylodiscitis has never been reported in previous studies.

Therefore, in this study, patients with single‐level lumbar spondylodiscitis that were treated with Mis‐OLIF without anterior or posterior instrumentation were retrospectively reviewed. The purpose of this study was to: (i) describe Mis‐OLIF strategy without anterior or posterior instrumentation for the treatment of single‐level lumbar spondylodiscitis; (ii) evaluate the efficacy and feasibility of Mis‐OLIF without anterior or posterior instrumentation in treating lumbar spondylodiscitis; and (iii) analyse clinical fusion effect of iliac graft for the treatment of lumbar spondylodiscitis. We found that Mis‐OLIF without anterior or posterior instrumentation and iliac graft is an effective and viable approach for the treatment of conservatively ineffective lumbar spondylodiscitis without spinal deformity or epidural abscess.

## Materials and Methods

### *Inclusion and Exclusion Criteria*

The inclusion criteria included: (i) diagnosis of single‐level spondylodiscitis without spinal deformity or epidural abscess; (ii) ineffective conservative treatment (continuously aggravated clinical symptoms and uncontrollable infective symptoms treated with antibiotics for more than 6 weeks); (iii) Mis‐OLIF and iliac graft for the treatment of lumbar spondylodiscitis; (iv) postoperative follow‐up >12 months; and (v) retrospective study. Exclusion criteria were as follows: (i) lumbar infections after other lumbar surgeries; (ii) treatment with other surgical approaches; and (iii) lumbar trauma.

### *Patient Data*

A retrospective study was performed between December 2016 and November 2017 for 14 consecutive patients with single‐level lumbar spondylodiscitis treated with Mis‐OLIF without anterior or posterior instrumentation. The characteristics of patients and individual data, including age, gender, surgical segments, clinical symptoms, radiographic data, surgical approach, operating duration, blood loss, follow‐up, pathogens, and the American Spinal Injury Association (ASIA) neurological classification, for the 14 patients are shown in Table 1.

**Table 1:** Demographic characteristic of patients

Patient	Gender	Age (years)	Segment	Clinical symptoms	Radiological data	Surgical approach	Operating duration	Blood loss	Follow‐up	Pathogens	ASIA classification		
											Pre‐operation	Before discharge	Final follow‐up
1	M	48	L3/4	Low back pain, pain in both lower extremities	Bone defect, paravertebral abscess	OLIF	70	120	12	Enterobacterium	D	E	E
2	M	52	L3/4	Low back pain	Bone defect	OLIF	90	150	24	Brucellosis	E	E	E
3	M	44	L3/4	Low back pain with progressive aggravation	Bone defect	OLIF	60	90	22	Brucellosis	E	E	E
4	F	45	L3/4	Low back pain, fever, weakness of right lower extremity	Bone defect, paravertebral abscess	OLIF	80	140	15	Brucellosis	D	D	E
5	M	49	Ll/2	Low back pain, pain and weakness in right lower extremity	Bone defect	OLIF	90	200	18	Staphylococcus aureus	D	D	D
6	M	74	L4/5	Low back pain with limitation of motion	Bone defect, destruction of intervertbral space	OLIF	120	250	15	Undefined	D	D	E
7	M	45	L2/3	Low back pain, fever	Destruction of intervertbral space, paravertebral abscess	OLIF	90	140	12	Staphylococcus aureus	E	E	E
8	M	46	L3/4	Low back pain, pain in left lower extremity	Bone defect, destruction of intervertbral space	OLIF	100	250	15	Staphylococcus aureus	E	E	E
9	F	51	L4/5	Low back pain with progressive aggravation, weakness of both lower extremities	Bone defect, paravertebral abscess	OLIF	90	160	24	Brucellosis	D	D	D
10	F	42	L2/3	Low back pain, fever, weakness, of both lower extremities	Bone defect, paravertebral abscess	OLIF	90	100	16	Staphylococcus aureus	D	D	E
11	F	43	L3/4	Low back pain with progressive aggravation	Bone defect, psoas abscess	OLIF	90	120	20	Enterobacterium	E	E	E
12	F	49	L4/5	Low back pain with progressive aggravation	Bone defect	OLIF	120	200	12	Brucellosis	E	E	E
13	M	42	L3/4	Low back pain	Bone defect, destruction of intervertbral space	OLIF	100	130	18	Brucellosis	E	E	E
14	F	57	L3/4	Low back pain with progressive aggravation	Bone defect, destruction of intervertbral space	OLIF	60	120	12	Staphylococcus aureus	E	E	E
Average ± SD	—	49.1 ± 8.0	—	—	—	—	89.3 ± 17.5	155.0 ± 49.4	16.8 ± 4.2	—	—	—	—

ASIA, American Spinal Injury Association; OLIF, oblique lumbar interbody fusion; SD, standard deviation.

### *Preoperative Evaluation*

For an accurate diagnosis, the X‐ray, CT, and MRI were performed in all patients. The preoperative laboratory examinations, including blood routine examination, ESR, CRP, procalcitonin, bacterial culture, hepatorenal function, and other routine laboratory examinations provided effective diagnostic references. For four of the patients using needle biopsies of pathological vertebrae, a conclusive diagnosis was made, but the specific pathogen in one of the patients was not confirmed. Antibiotic treatment was principally based on the results of bacterial culture, and secondly on the clinical experience. Patients received irregular anti‐infection therapy prior to admission and preoperative antibiotic therapy for approximately 2 weeks in our hospital. In this study, the indications of Mis‐OLIF surgery were ineffective conservative antibiotic treatment (approximately >6 weeks), the existence of paravertebral abscess or sequestrum, and persistent and unmitigated low back pain with or without neurological symptoms.

### *Surgical Procedure of Mis‐OLIF*

**Figure 1:**
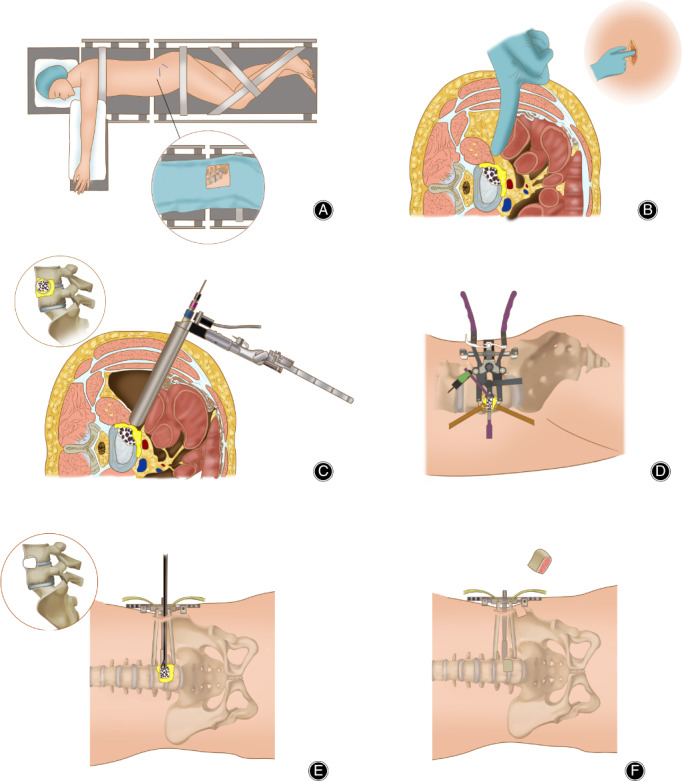
Surgical procedures of minimally invasive surgery oblique lumbar interbody fusion. (A) Patient position and surgical incisions; (B) Blunt dissection of muscles was performed to approach to retroperitoneal intermuscular space; (C and D) The sequential dilators and the retractor was placed and fixed for visualizing the infective focus; (E) Debridement of infective focus; (F) Autologous iliac bone was implanted into the intervertebral space.

The patients were placed in the right lateral decubitus position after general anesthesia was administered. A 4‐cm long incision parallel to the musculus obliquus externus abdominis was made in the left lateral abdominal region, and a blunt dissection of the musculus obliquus externus and internus abdominis and musculus transversus abdominis was performed to approach the retroperitoneal intermuscular space. After clearing the retroperitoneal space and palpating the psoas, initial dilator was inserted to the intervertebral space and positioned by fluoroscopy. Then the sequential dilators were introduced and the retractor of OLIF was placed and fixed. The infective focus, including infective abscesses, necrotic vertebras, and intervertebral disc, were completely removed. After the debridement, autologous iliac bone of appropriate size was implanted into the intervertebral space. Autologous iliac bone graft was taken from the left anterosuperior iliac spine, and the size of iliac bone graft was based on the size of bone defect. These procedures were presented in Fig. 1.

### *Postoperative Management*

**Figure 2:**
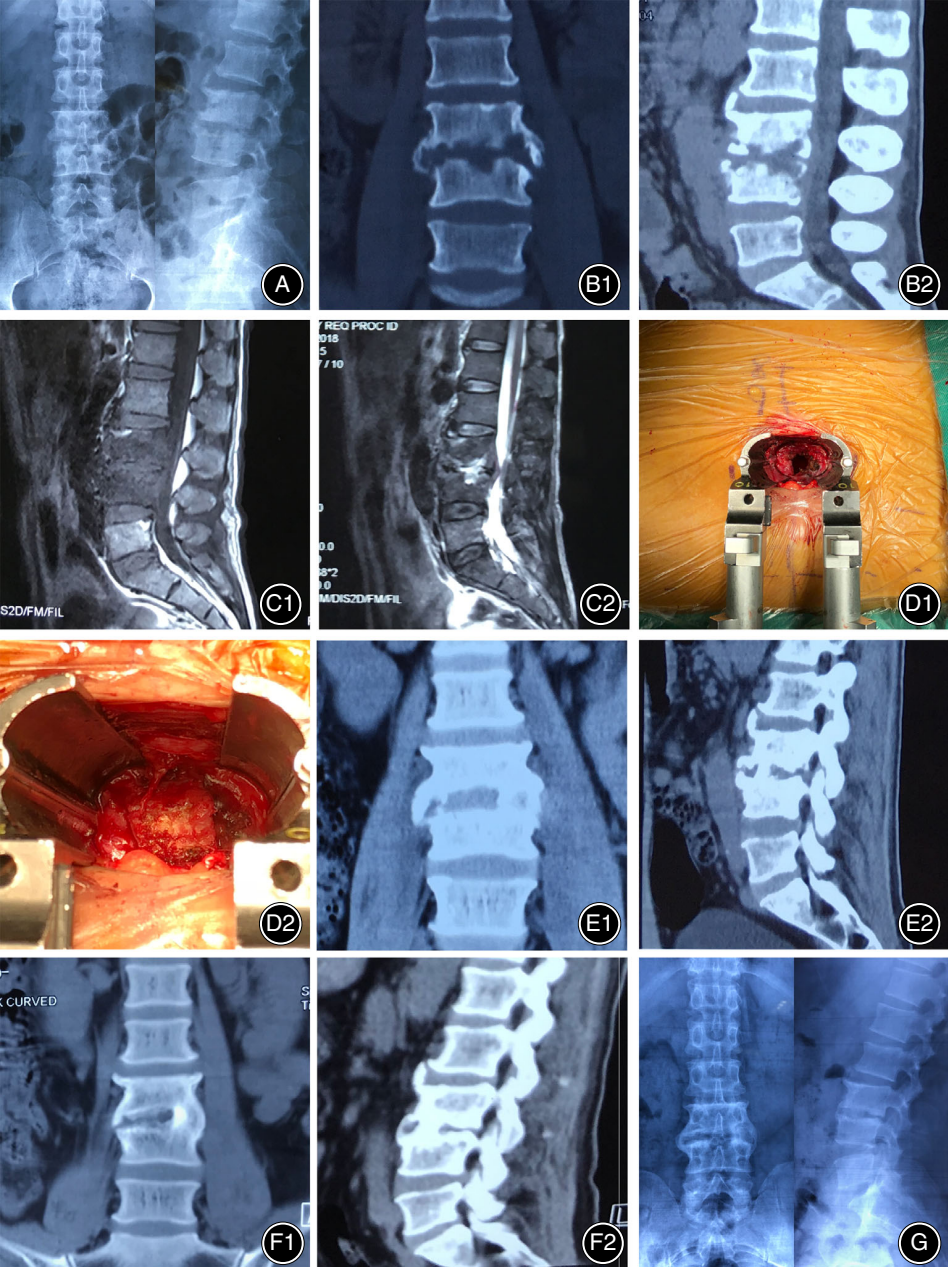
Forty‐eight‐year‐old man, whose complaint was low back and right lower limb pain of more than 2 months. Minimally invasive surgery oblique lumbar interbody fusion (Mis‐OLIF) was performed. Bone defect and paravertebral abscess were seen in the preoperative images and during surgery. (A) preoperative plain radiographs of lumbar; (B1 and B2) preoperative CT images; (C1 and C2) preoperative MRI; (D1 and D2) intraoperative pictures; (E1 and E2) CT images before discharge; (F1 and F2) CT images at 6 months follow‐up; (G) plain radiographs at 6 months follow‐up.

**Figure 3:**
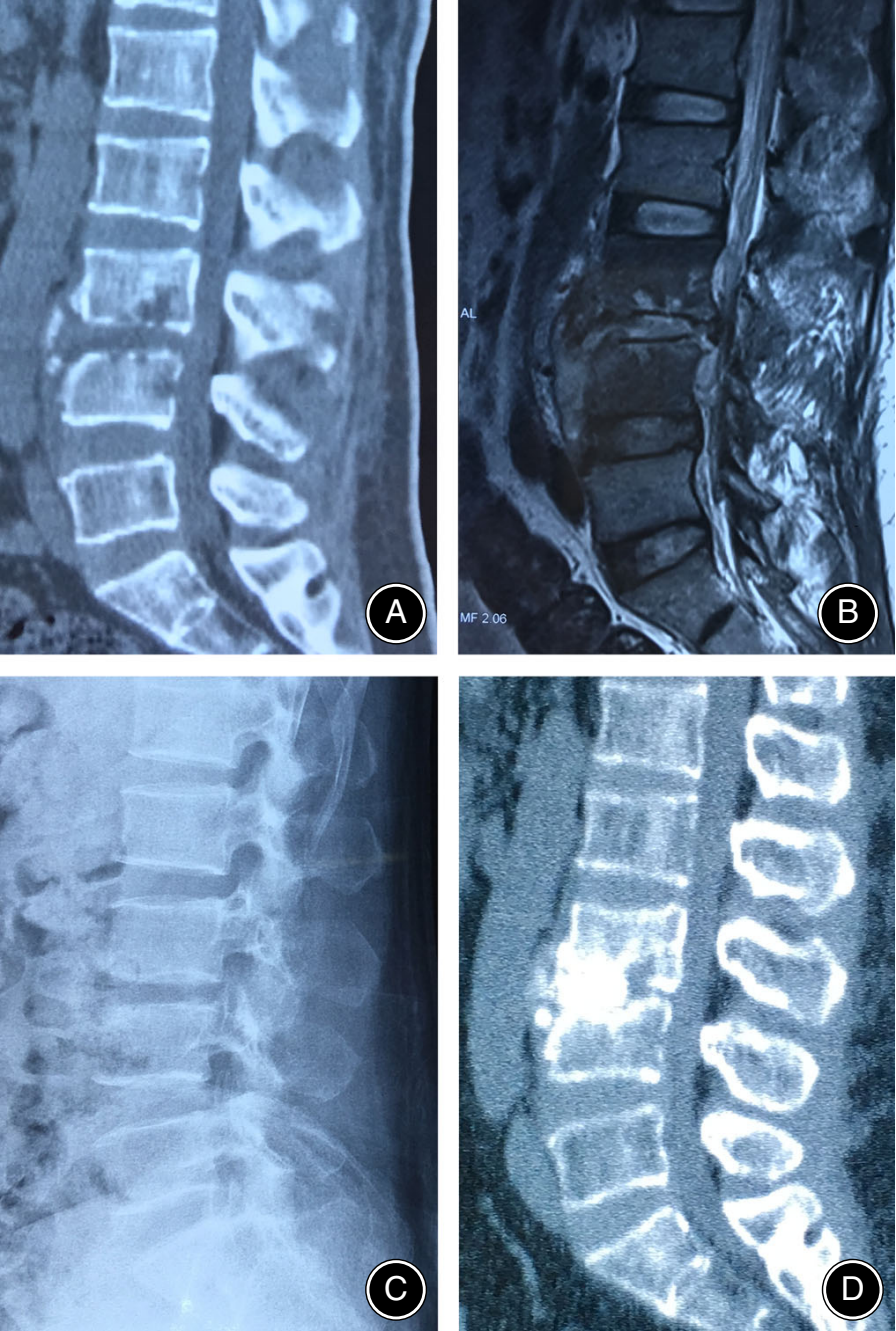
Preoperative CT (A) and MRI (B) images revealed L3/4 intravertebral space infection with endplate destruction; At 1 year of follow‐up, both the X‐ray (C) and CT images (D) showed bony fusion between bone graft and vertebrae interface.

**Figure 4:**
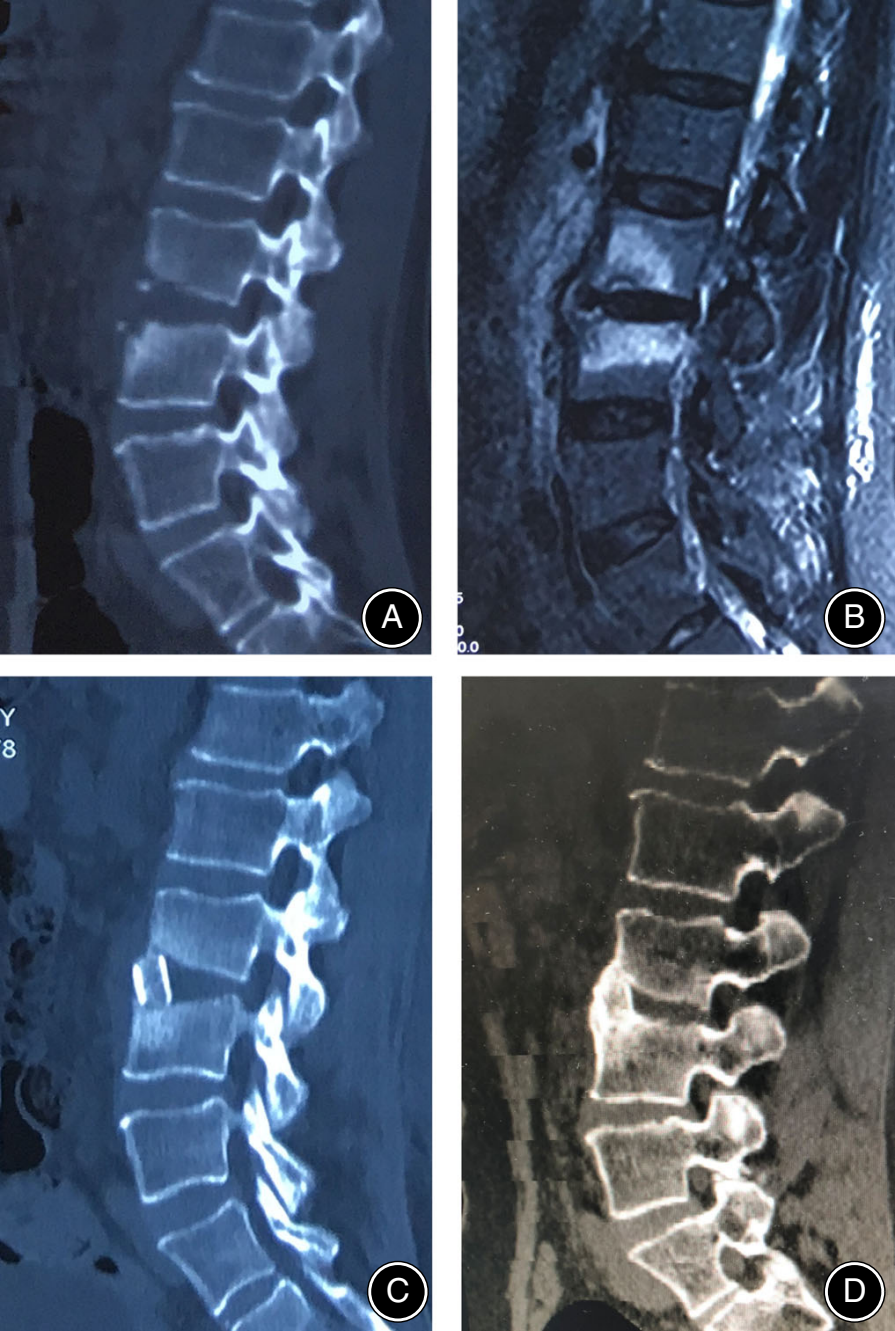
Preoperative CT (A) and MRI (B) images revealed L3/4 intravertebral space infection with superior endplate destruction. A massive structure iliac graft was seen in CT scan (C) before discharge. One year after Mis‐OLIF, CT scan (D) showed bony fusion between bone graft and vertebrae interface.

Thoracolumbosacral bracing was necessary to maintain the location of autologous iliac bone and help to achieve bony fusion. Patients received postoperative antibiotic therapy for approximately 2–4 weeks before discharge. Antibiotic therapy continued for approximately 4 weeks after discharge, and the antibiotic medication was adjusted according to the blood routine examination, ESR, and CRP during the 1‐month follow‐up. The fusion rates were evaluated by the X‐ray and CT scans at final follow‐up 20, 21. Figure 2 shows the preoperative, postoperative radiological images and intraoperative pictures of a patient who was treated with Mis‐OLIF. Figures 3, 4 show the preoperative CT and MRI images and bony fusion at follow‐up of the patients who were treated with Mis‐OLIF.

### *Outcome Measures*

#### *Visual Analogue Scale*

The Visual Analogue Scale (VAS) scoring system was used to evaluate the pain level of patients. The VAS scoring system is self‐completed by the patient. Patients mark the location on the 10‐cm line corresponding to the amount of pain they experienced. 0 is no pain and 10 is severest pain.

#### *Oswestry Disability Index*

Oswestry Disability Index (ODI) is a principal condition‐specific outcome measure used in the management of spinal disorders, and to assess patient progress in routine clinical practice. The ODI score system includes 10 sections: pain intensity, personal care, lifting, walking, sitting, standing, sleeping, sex life, social life, and traveling. For each section of six statements, the total score is 5. Intervening statements are scored according to rank. If more than one box is marked in each section, take the highest score. If all 10 sections are completed the score is calculated as follows: total score out of total possible score × 100. If one section is missed (or not applicable), the score is calculated: (total score / (5 × number of questions answered)) × 100%. Scores are as follows: 0%–20% is considered mild dysfunction; 21%–40% is moderate dysfunction; 41%–60% is severe dysfunction; and 61%–80% is considered as disability. For cases with score of 81%–100%, the patient is either long‐term bedridden, or exaggerating the impact of pain on their life.

#### *American Spinal Injury Association Score*

The American Spinal Injury Association Score (ASIA) is commonly used to evaluate neurological deficits. It is based on motor and sensory scores, neurological levels, a completeness criterion, zones of partial preservation, and an impairment scale. The impairment scale is as follows: (A) no sensory or motor function; (B) incomplete sensory but no motor function; (C) incomplete motor function is preserved below the neurological level and more than half of the key muscles below the neurological level have a muscle grade less than 3; (D) incomplete motor function is preserved below the neurological level, and more than half of the key muscles below the neurological level have a muscle grade greater than or equal to 3; and (E) sensory and motor function are normal.

### *Complications*

Postoperative complications, including subsidence, ileus, vascular injury, nerve injury, ureteral injury, incision‐related complication, and fusion‐related complication were recorded and analyzed.

### *Statistical Analysis*

The preoperative, postoperative, and final follow‐up ESR, CRP, VAS scores and ODI were analyzed using SPSS 25.0 software (SPSS, Chicago, Illinois, USA). The paired t‐test was used to assess the difference between the preoperative, postoperative, and final follow‐up ESR, CRP, VAS scores and ODI. *P* values <0.05 was considered statistically significant.

## Results

### *General Results*

From December 2016 to November 2017, 14 consecutive patients (eight males; six females) with lumbar spondylodiscitis were included. The mean age of the patients at the time of treatment was 49.1 ± 8.0 years (range, 42–74 years). The mean follow‐up was 16.8 ± 4.2 months (range, 13–24 months).

All patients underwent Mis‐OLIF without any instrumentation. The mean duration of surgery was 89.3 ± 17.5 min (range, 60–120 min). The mean blood loss was 155.0 ± 49.4 mL (range, 90–250 mL). The results and statistics are shown in Table 1.

The infectious levels included L1/2 (1), L2/3 (2), L3/4 (8), and L4/5 (3). Intraoperative pus cultures were obtained for all 14 patients. The pathogens found in these patients included Staphylococcus aureus (five patients), brucellosis (six patients), and enterobacterium (two patients). In one of the patients, the pathogen was undefined, even though the clinical symptoms, radiological findings, laboratory, and pathological examinations were all combined to achieve a diagnosis.

### *Clinical Improvement*

Clinical symptoms including low back pain, fever, and weakness or pain of lower extremity improved after surgery and significantly improved at the final follow‐up. Infective indicators (ESR and CPR) decreased to normal levels at the final follow‐up.

### *ESR*

**Figure 5:**
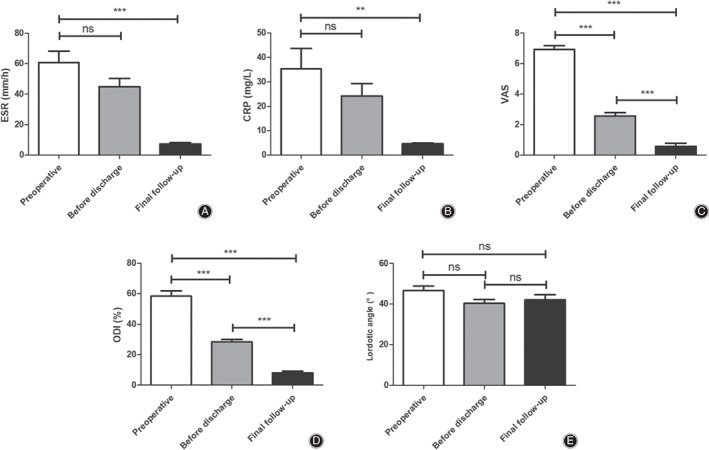
A‐E Comparison of ESR, CRP, VAS, ODI, and Lordotic angle preoperatively, before discharge, and at final follow‐up. Ns, not significant, **P < 0.01; and ***P < 0.001.

In Table 2 and Fig. 5, the ESR decreased after Mis‐OLIF and antibiotic treatment. The ESRs preoperatively, before discharge, and at final follow‐up were 60.8 ± 27.1 mm/h, 44.9 ± 20.0 mm/L, and 7.4 ± 3.2 mm/h, respectively.

**Table 2:** Characteristics and clinical data of the patients (mean ± SD)

Variable	Preoperative	Before discharge	Final follow‐up
ESR (mm/h)	60.8 ± 27.1	44.9 ± 20.0	7.4 ± 3.2
CRP (mg/L)	35.3 ± 30.6	24.1 ± 18.7	4.7 ± 1.2
VAS	6.9 ± 0.9	3.0 ± 1.0	0.6 ± 0.7
ODI (%)	58.4 ± 13.0	28.3 ± 6.1	8.0 ± 4.6
Lordotic angle (°)	46.6 ± 8.0	40.4 ± 6.9	42.0 ± 9.3

However, there was no significant difference between preoperative ESR and before discharge (from 60.8 ± 27.1 mm/h to 44.9 ± 20.0 mm/L, t = 2.08, *P* > 0.05). Compared with preoperative ESR, it significantly decreased at final follow‐up (from 60.8 ± 27.1 mm/h to 7.4 ± 3.2 mm/h, t = 6.98, *P* < 0.05) (Fig. 5).

### *CRP*

In Table 2 and Fig. 5, the CRP decreased after Mis‐OLIF and antibiotic treatment. The CRPs preoperatively, before discharge, and at the final follow‐up were 35.3 ± 30.6, 24.1 ± 18.7, and 4.7 ± 1.2 mg/L, respectively.

However, there was no significant difference between preoperative CRP and before discharge (from 35.3 ± 30.6 to 24.1 ± 18.7 mg/L, t = 1.38, *P* > 0.05). Compared with preoperative CRP, it significantly decreased at the final follow‐up (from 35.3 ± 30.6 to 4.7 ± 1.2 mg/L, t = 3.78, *P* < 0.01) (Fig. 5).

### *VAS*

According to Table 2 and Fig. 5, the pain was significantly relieved according to VAS scores and the average VAS score was 6.9 ± 0.9 preoperatively, and decreased to 3.0 ± 1.0 (t = 14.18, *P* < 0.001) and 0.6 ± 0.7 (t = 20.68, *P* < 0.001) before discharge and at final follow‐up, respectively.

### *Radiographic Improvement*

According to Table 2 and Fig. 5, the Lordotic angle preoperatively, before discharge, and at final follow‐up was 46.6° ± 8.0°, 40.4° ± 6.9°, and 42.0° ± 9.3°, respectively.

As indicated in Fig. 5, the Lordotic angle decreased after Mis‐OLIF and no obvious angle lost observed at the final follow‐up. Comparing preoperative Lordotic angle (46.6° ± 8.0°) with the angles before discharge (40.4° ± 6.9°) and at the final follow‐up (42.0° ± 9.3°), there was no significant difference (t = 8.81 before discharge and t = 14.77 at the final follow‐up, *P* > 0.05).

### *Implants Evaluation*

The location of autologous iliac bone had no obvious change at the final follow‐up, except for one patient who had subsidence of autologous iliac bone before discharge. All 14 patients achieved bony fusion at the final follow‐up.

Three typical cases with lumbar spondylodiscitis treated with Mis‐OLIF were selected. The preoperative radiological images, intraoperative situation of one of the cases, and bony fusion achieved at the final follow‐up according to plain radiographs and CT scans are shown in (Figs 2, 3, 4).

### *Functional Evaluation*

#### *ODI*

Functional disability was improved according to Table 2 and Fig. 5. The average ODI score was 58.4 ± 13.0% preoperatively, and decreased to 28.3 ± 6.1% (t = 18.6, *P* < 0.001) and 8.0 ± 4.6% (t = 22.7, *P* < 0.001) before discharge and at final follow‐up, respectively.

#### *Neurological Evaluation*

Neurological evaluation was assessed in Table 1, eigth patients had normal neurology (ASIA E). Four ASIA D patients preoperatively improved to ASIA E at the final follow‐up. Two patients with ASIA D preoperatively did not show neurologic recovery at the final follow‐up.

### *Complications*

None of the patients developed postoperative ileus, vascular injury, nerve injury, and ureteral injury.

However, one patient suffered incision‐related complication after surgery that healed by debridement and dressing change. One patient developed subsidence of autologous iliac bone before discharge and achieved complete bony fusion after staying in bed and fixing it with a brace at 3 months follow‐up.

## Discussion

Spondylodiscitis mostly affects the lumbar spine, followed by the thoracic and cervical spine^22^, ^23^, ^24^, ^25^. The most common clinical symptom of lumbar spondylodiscitis is low back pain, followed by fever^4^, ^24^. The most serve influence of spondylodiscitis is death, and patients without a standard and effective treatment are more likely to relapse. Previous studies reported that spinal spondylodiscitis is mostly caused by *Staphylococcus aureus* 4, 22. In our study, *Staphylococcus aureus* and brucellosis are the main pathogens in lumbar spondylodiscitis. Moreover, lumbar spondylodiscitis mostly affects the L3/4 level.

The purpose of treatment in spondylodiscitis is to cure the infection, release the symptoms, and restore spinal stability. Treatment methods for spinal spondylodiscitis remain controversial. It has been reported that a 6‐week antibiotic treatment is sufficient for most patients with nonspecific spondylodiscitis^4^. However, a multicenter observational prospective study showed that the mean antibiotic treatment duration for non‐tuberculosis spondylodiscitis was 12.4 weeks^26^. Antibiotic treatment has proved to be sufficient and effective in many studies, but is not always successful^9^, ^22^, ^27^. Early surgery with antibiotic treatment could reduce hospital stays and dosage of antibiotics^9^. Moreover, conservative treatment increases the risk of deformity in the long term, perioperative complications, and surgical outcomes are poorer in patients with delayed surgical treatment^22^. Due to the heterogeneity of spondylodiscitis, diversity of pathogens, and increasing resistance of pathogens, less invasive surgical procedures are possibly preferred in the early stages of infection and in patients of doubt or progression^22^. Moreover, it is difficult to achieve treatment consensus.

Surgery is performed due to spinal instability, local abscesses, compression of the nerve or spinal cord, and spinal deformity^2^, ^4^, ^23^, ^24^, ^28^, ^29^, ^30^. The types of surgery include CT‐guided percutaneous drainage for abscess, anterior decompression, posterior laminectomy and decompression, and anterior decompression with posterior instrumentation^10^, ^12^, ^25^, ^29^, ^30^, ^31^. A report suggested that CT‐guided percutaneous drainage could be effective for selective patients with spondylodiscitis and secondary abscess^31^. Posterolateral endoscopic surgery for lumbar spondylodiscitis brought good clinical results^28^. Patients with posterior stabilization treated with anterior decompression and bone graft achieved best clinical outcomes^25^. It was demonstrated that posterior decompression and fusion could lead to good results in patients with hematogenous lumbar spondylodiscitis^30^. A report concluded that anterior decompression with delayed posterior instrumentation was an option for spinal spondylodiscitis^29^. Extreme lateral interbody fusion with posterior instrumentation may be an effective alternative to anterior lumbar interbody fusion (ALIF) for the treatment of spondylodiscitis^32^. Mis‐OLIF as an effective approach for the treatment of degenerative lumbar diseases has been widely applied^16^, ^18^, ^33^, ^34^. In our study, the purpose is to evaluate the efficacy and feasibility of Mis‐OLIF without anterior or posterior fixation in the treatment of lumbar spondylodiscitis without spinal deformity or epidural abscess.

### *Clinical Effectiveness of Mis‐OLIF*

Regarding clinical outcomes, the clinical symptoms evidently improved in our study, according to the VAS and ODI scores. Even though the ESR and CRP before discharge showed no significant improvement compared with preoperative values, there was a declining trend, and the ESR and CRP returned to normal at follow‐up. The possible reasons could be stress reaction to surgery and the normal course of the disease. In addition, the patients in our study were diagnosed as lumbar spondylodiscitis without spinal deformity or epidural abscess and the lumbar vertebral bodies were mildly destroyed. Therefore, this may explain why there are no significant differences between the preoperative Lordotic angle and postoperative ones. The main purposes of surgery for lumbar spondylodiscitis include curing the infection and releasing the symptoms^22^. A previous study reported the efficiency and safety of the OLIF corridor approach in treating lumbar pyogenic spondylodiscitis^19^. Similarly, in this study, Mis‐OLIF can relieve the symptoms of patients with lumbar spondylodiscitis, improve the diagnostic efficiency and treatment, and achieve good bony fusion. OLIF corridor approach could achieve complete debridement, because the lesion can be clearly viewed and debrided precisely.

### *Spine Stability and Effective Bony Fusion*

For patients with lumbar spondylodiscitis, anterior or posterior fixation may be necessary due to the spine being unstable^10^, ^35^. However, it was unnecessary to equip the patients with instrumentations, due to mild destruction of lumbar vertebral bodies. In this study, lumbar debridement and fusion surgery should be performed on patients mainly because of ineffective conservative treatment. The iliac bone graft needed in the surgery was relatively small due to mild destruction. The complete debridement is not only to help relieve symptoms and prevent recurrence, but also to benefit better bony fusion. The purpose of the iliac bone graft was to maintain stability of the anterior column and achieve good bony fusion. The results showed that all patients achieved bony fusion at follow‐up. This indicated that Mis‐OLIF with iliac bone graft is effective and sufficient to achieve bony fusion. In addition, without the anterior or posterior fixation, the iliac bone grafts could only improve spinal anterior column stability and achieve bony fusion.

### *Complications*

The common complications that occurred in OLIF surgery include subsidence, postoperative ileus, vascular injury, nerve injury, ureteral injury, and blood transfusion^17^, ^18^, ^33^. CT‐guided percutaneous drainage has limited indications^31^. The disadvantage of posterolateral endoscopic surgery is the potential to disturb the spinal canal, thereby increasing the risk of intraspinal infection. The incidence rate and severity of complications of OLIF are much lower than those of ALIF^17^, ^29^. Compared to ALIF with posterior percutaneous fixation in the treatment of single‐level lumbar spondylodiscitis, mean blood loss and duration of surgery in our study are lesser than those reported in previous study, and the complication of urinary injury can be avoided^36^. In this study, one patient suffered incision‐related complication and another developed subsidence of autologous iliac bone. The incidence of complications is likely acceptable in contrasting with other surgeries. Mis‐OLIF has the advantages of less duration of surgery, less blood loss, less tissue trauma, and avoiding disturbance of the spinal canal. In this study, no significant intraoperative complication was observed during surgery, which suggests that the Mis‐OLIF is safe for managing lumbar spondylodiscitis. Moreover, the unspecific clinical manifestations can lead to a delay in diagnosis of spondylodiscitis, and this may explain the mortality of spinal spondylodiscitis. Mis‐OLIF can be performed in the early stages of lumbar spondylodiscitis. Therefore, Mis‐OLIF not only plays a curative role, but also helps in earlier diagnosis.

### *Limitations*

Our study has some limitations such as it being a small‐size retrospective study with a lack of control groups. The effectiveness of OLIF in multi‐level lumbar spondylodiscitis is unclear. Moreover, OLIF is usually performed from the left side, and further study is warranted to confirm the effectiveness of Mis‐OLIF to manage psoas abscess on the right side. We believe that a multi‐center, longer follow‐up randomized control study is needed to ameliorate the limitations of this study. Nonetheless, this study shows that Mis‐OLIF is a viable treatment option for lumbar spondylodiscitis.

### *Conclusion*

Mis‐OLIF without anterior or posterior instrumentation and the iliac graft is an effective and viable approach in the treatment of conservatively ineffective lumbar spondylodiscitis without spinal deformity or epidural abscess. It has achieved effective clinical and radiological outcomes. Mis‐OLIF reduces clinical manifestations, improves the diagnostic efficiency and treatment, and achieves good bony fusion. Additionally, it may reduce operation time, blood loss, and tissue trauma, thus avoiding disturbance of the spinal canal.
